# Factor structure of the parental reflective functioning questionnaire and association with maternal postpartum depression and comorbid symptoms of psychopathology

**DOI:** 10.1371/journal.pone.0254792

**Published:** 2021-08-02

**Authors:** Katrine I. Wendelboe, Johanne Smith-Nielsen, Anne C. Stuart, Patrick Luyten, Mette Skovgaard Væver

**Affiliations:** 1 Department of Psychology, Center for Early Intervention and Family Studies, University of Copenhagen, Copenhagen, Denmark; 2 Faculty of Psychology and Educational Sciences, KU Leuven, Leuven, Belgium; 3 Research Department of Clinical, Educational and Health Psychology, University College London, London, United Kingdom; 4 Yale Child Study Center, Yale University School of Medicine, New Haven, Connecticut, United States of America; Unviersity of Heidelberg, GERMANY

## Abstract

Parental reflective functioning (PRF) refers to the parent’s capacity to envision mental states in the infant and in themselves as a parent, and to link such underlying mental process with behavior, which is important for parenting sensitivity and child socio-emotional development. Current findings have linked maternal postpartum depression to impaired reflective skills, imposing a risk on the developing mother–infant relationship, but findings are mixed, and studies have generally used extensive methods for investigating PRF. The present study examined the factor structure and measurement invariance of the Danish version of the 18-item self-report Parental Reflective Functioning Questionnaire (PRFQ) in a sample of mothers with and without diagnosed postpartum depression. Moreover, the association between PRF and maternal postpartum depression in mothers with and without comorbid symptoms of personality disorder and/or clinical levels of psychological distress was investigated. Participants included 423 mothers of infants aged 1–11 months. Confirmatory factor analysis supported a three-factor structure of the PRFQ; however, item loadings suggested that a 15-item version was a more accurate measure of PRF in mothers of infants. Multi-group factor analysis of the 15-item PRFQ infant version indicated measurement invariance among mothers with and without diagnosed postpartum depression. Multinomial logistic regression showed that impaired PRF was associated with maternal psychopathology, although only for mothers with postpartum depression combined with other symptoms of psychopathology. These results provide new evidence for the assessment of maternal self-reported reflective skills as measured by a modified infant version of the PRFQ, as well as a more nuanced understanding of how variance in symptomatology is associated with impaired PRF in mothers in the postpartum period in differing ways.

## Introduction

Parental reflective functioning (PRF), or parental mentalizing, is defined as the parent’s ability to reflect upon the ongoing psychological processes in their child and in themselves as a parent [1]. Thus, PRF allows the parent to “decode” infant behavior, linking it to internal states, and thereby provides the parent with a greater understanding of the infant’s needs [1]. PRF is considered to play a role in parental sensitivity [2] and in important aspects of child development such as emotion regulation and the development of the child’s own reflective skills, as well as child behavioral problems [3–5]. Moreover, PRF is proposed to be a relationship-specific aspect of mentalizing [6,7], and the emerging relationship between parent and infant is considered to be a special one, as it holds unique demands for the parent’s reflective skills due to infant communication being limited to non-verbal signals and the parent’s role in regulating the infant [8,9]. Additionally, studies have shown that PRF, like mentalizing in general, is multidimensional and that individuals with different types of psychological problems demonstrate imbalances between different dimensions of mentalizing [10,11]. This also implies that in parents some dimensions of PRF might be intact, while contextual factors and/or parental psychopathology might affect other dimensions [12,13].

Impairments in PRF typically lead to problems in adequately “reading” the child’s mind, which, in turn, negatively influence the quality of parental caregiving behavior [14–16]. Parental psychopathology, such as depression, has been found to compromise reflective capacities [17], and recently the effect of maternal postpartum depression (PPD) on PRF has become an important area of investigation within the field of parent and child mental health research [18].

PRF has primarily been assessed in research and clinical settings using semi-structured interviews such as the Reflective Functioning Scale [19] for the Parent Development Interview [20], which is considered a gold standard measure of PRF, and the Working Model of the Child Interview [21]. While well-validated and clinically informative, such measures are cost-intensive and, for this reason, their use in large-scale research and clinical settings has been limited. Furthermore, these measures yield a total PRF score, which may not fully capture the proposed multidimensionality of PRF.

One measure developed to capture the multidimensional aspect of PRF is the Parental Reflective Functioning Questionnaire (PRFQ) [22]. The PRFQ is a self-report questionnaire for parents of young children (age 0–5 years) tapping into three different dimensions of parental reflective skills: Prementalizing (PM), reflecting a non-mentalizing stance towards infant mental states and distorted interpretations, Interest and Curiosity (IC) in infant mental states, and Certainty about Mental States (CMS), reflecting an inability to recognize the opacity of mental states or to be overly doubtful regarding interpretations [23]. Since the development of the PRFQ, its dimensional structure has been examined in different studies. Mousawi et al. [24] found evidence for the three-factor structure of the Iranian version of the PRFQ using exploratory factor analysis in a sample of 244 mothers of 1–5-year-old children. In another study, confirmatory factor analysis (CFA) of the Italian version of the PRFQ demonstrated good model fit of the proposed three-factor structure in a sample of 385 mothers and fathers of children aged 3–10 years [25]. Likewise, in a sample of 306 Canadian parents (n = 186 mothers) of 0–12-year-old children, De Roo et al. [26] found that CFA indicated an acceptable model fit of the three-factor structure of the PRFQ, although some revision was needed for optimal fit, namely omitting item 11 (“I can sometimes misunderstand the reactions of my child.”) and item 18 (“I believe there is no point in trying to guess what my child feels”). However, while the PRFQ is being increasingly used in studies [24–31], few have investigated its factor structure in clinical samples and in parents of primarily young infants. Replication of factor structure, including examination of measurement invariance, is an important prerequisite for investigating potential differences among study subjects, especially when assessing psychological constructs that can have different meanings to different groups, such as PRF [32]. This study therefore sets out to examine the factor structure of the PRFQ and invariance among mothers with and without diagnosed PPD. Secondly, this study investigates potential impairments of PRF in relation to maternal PPD with and without comorbid psychopathology symptoms.

### Maternal reflective functioning, postpartum depression and comorbidity

Extensive research has documented how depression compromises psychological functioning [33,34]. In particular, social cognition has been found to be impaired in depressed individuals [35], and researchers have focused on deficits in mentalizing as a potential explanation for this [36–38]. From a mentalizing theory perspective, cognitive distortions regarding the self and others may be understood as mentalizing impairments, resulting from stress responses to perceived threats to the person’s attachment system and sense of self [39].

In the postpartum period, the prevalence of depression is up to 17% even in mothers with no prior history of mental disorders [40], and PPD is considered to be one of the most disabling conditions due to its impact on maternal psychological wellbeing [41] and maternal caregiving behavior, as well as on child outcomes [42–46]. Concerning the association between maternal depressive symptoms and mentalizing skills in the postpartum period, the findings are not uniform. While some studies find that depression is indeed associated with impaired maternal mentalizing skills [14,47–49] other studies find mixed [50] to non-significant [51,52] associations. The inconsistency among the findings may be due to differences in the conceptualization and measurement of PRF. Furthermore, in most of the studies mothers’ depressive symptoms have been assessed by screening instruments and not by using a diagnostic interview. As evidence suggests that impairments in mentalizing are more pronounced when depression is severe [17,53], it is possible that the depressive symptoms in many cases included in these studies [14,49–52] are more transient and less pervasive than in the case of a diagnosed clinical depression.

The mixed findings also question whether PPD *per se* is associated with impaired maternal reflective functioning, or whether other coexisting psychological problems may play a role. This is supported by literature suggesting that mothers with PPD are a heterogeneous group, potentially struggling with different underlying psychological disturbances [54]. Attention has therefore been brought to other aspects of psychological problems affecting mothers’ postpartum parenting skills, such as personality disorders (PDs) [55] and overall psychological distress not limited to a specific disorder [56].

Studies indicate that mothers with PD are more likely to develop depressive symptoms in the postpartum period than mothers without PD [57,58]. Literature on PD in relation to motherhood has demonstrated how the condition adversely affects the mother–infant interaction in several ways, which constitutes a risk for child socio-emotional wellbeing [59–61]. Although PD has been linked with impaired mentalizing skills [62–64], studies on mentalizing in mothers with PD, particularly in the postpartum period, are still scarce and results have been mixed, with significant [65,66] to non-significant [67] associations.

Given that many psychiatric conditions are comorbid, and that patients may replace one symptom with another over time, it has been suggested that non-specific emotional distress is an appropriate indicator of psychosocial functioning [68–71]. Indeed, researchers have highlighted a need for a broader focus on psychological disturbances and general symptom severity in the postpartum period and indications thereof, arguing that maternal PPD alone does not necessarily function as the primary marker for mental health and potential problematic parenting behaviour [72,73]. Accordingly, evidence indicate that non-specific maternal psychological distress can have negative consequences for the mother–infant relationship and child development [74,75], yet only few studies have examined the association between psychological distress and mentalizing skills [76,77], and fewer have focused on PRF specifically.

In sum, findings on PRF in mothers with depression have been mixed. This may be due to differences in methodology, but it might also be a function of the heterogeneity of mothers with PPD, and the lack of taking into account symptoms of other comorbid psychopathology and overall indicators of non-specific symptom severity.

### The present study

The objectives of this study are twofold. First, we investigate the factor structure of the PRFQ in a Danish at-risk sample of mothers with infants aged 1–11 months using CFA. Previous factor analyses of the PRFQ [23–26] proposed a three-factor model consisting of 1) interest and curiosity, 2) prementalizing and 3) certainty about mental states. We also conduct a multi-group CFA to test configural, metric and scalar invariance between mothers with subclinical levels of PPD and mothers with clinical PPD as assessed by a diagnostic interview.

Second, we aim to investigate associations between maternal PRF as measured by the PRFQ and PPD, with or without symptoms of comorbid PD and with or without clinical levels of psychological distress. Specifically, we investigate associations between 1) PRF and a diagnosis of PPD, 2) PRF and a diagnosis of PPD in combination with symptoms of PD, 3) PRF and PPD in combination with clinical levels of psychological distress, and, finally, 4) PPD in combination with both symptoms of PD and clinical levels of psychological distress. Based on the literature reviewed, we expect that more severely impaired PRF on the PRFQ is related to a higher probability of the presence of PPD, and of PPD with comorbid symptomatology.

## Methods

### Procedure and sample

As part of the general health care system in Denmark, all families are offered home visits by public health visitors (nurses specialized in infant and child development and perinatal health) within the first year following childbirth. This study was conducted as part of the Copenhagen Infant Mental Health Project (CIMHP), a collaborative project with the health visitors from the municipality of Copenhagen and the Center for Early Intervention and Family Studies, University of Copenhagen, which evaluates the effect of an early screening and intervention programme [78]. Families were recruited from July 2015 until July 2019 by the public health visitors who, as part of standard health care practice, visited mothers at 2, 4 (only first-time mothers) and 8 months postpartum. At the 2-month visit, the health visitors routinely screen mothers for signs of PPD using the Edinburgh Postnatal Depression Scale (EPDS) [79]; however, some mothers were also screened at other time points based upon the clinical judgement of the health visitors. If the mother scored above the cut-off on the EPDS (≥10, range 0–30), the family was informed about the CIMHP and invited to participate. If the family was interested, a home visit with a clinical psychologist from the project was planned. During this visit, informed written consent was obtained and a diagnostic interview was conducted, as well as an interview assessing symptoms of PD. The mother also received an online survey, which included the PRFQ and a questionnaire assessing psychological distress. Thus, the present study is cross-sectional, as all data were collected at the time of the home visit. The project was approved by the Ethical Committee at the Department of Psychology, University of Copenhagen. At the time of approval, the committee consisted of Associate Professor Jan Nielsen, Professor MSO Barbara Hoff Esbjørn and Associate Professor Tone Roald (**Approval number:** 2015–10).

Participants for the present study were mothers included in CIMHP who had an EPDS screening above cut-off and who had completed the PFRQ. Exclusion criteria for participation in CIMHP were: bipolar I disorder or present psychotic disorder, severe intellectual impairment, present suicidal ideation and/or recent suicide attempt, present alcohol/substance abuse, and/or infant diagnosed with autism and/or early developmental delay prior to the health visitor’s screening. Four mothers fulfilled diagnostic criteria for bipolar I disorder as assessed by the psychologist at the home visit, and were thus excluded from the study. The final sample for the factor analysis consisted of 423 mothers, of whom 237 met diagnostic criteria for a current major depressive episode and 186 did not meet diagnostic criteria for current depression. Of the 423 mothers eligible for factor analysis, data were available for a subsample of 344 mothers for analyses on the relation between maternal psychopathology and PRF.

### Measures

The PRFQ [23] was used to assess PRF. It is a self-report questionnaire measuring parents’ reflective functioning concerning their child age 0–5 years. The measure consists of 18 statements about the child that correspond to one of three dimensions of PRF: 1) interest and curiosity in the child’s mental state (IC) (e.g. “I like to think about the reasons behind the way my child behaves and feel”), 2) the degree of certainty about the child’s mental state (CMS) (e.g. “I can completely read my child’s mind”) and 3) prementalizing (PM), i.e. difficulties recognizing the child’s mental state, including simplistic attributions or ascribing manipulative intentions to the child’s behaviour (e.g. “My child cries around strangers to embarrass me”). Statements are rated on a 7-point Likert scale from “completely agree” to “completely disagree”. In their paper, Luyten et al. [23] describes how scores indicating adequate PRF might differ depending on the sample characteristics and the specific PRFQ scale. For PM, lower scores are the most optimal; however, regarding IC and CM, very high scores might indicate hypermentalizing, that is, a form of highly cognitive pseudomentalizing causing over-interpretation of mental states [23]. Recent studies on the PRFQ have revealed a three-factor structure of the PRFQ [23,25].

The Structural Clinical Interview for DSM-V Axis I, research version (SCID-5, RV) [80] was used to diagnose current depression and to assess suicidal ideation and bipolar and psychotic symptoms. Trained SCID-5 interviewers, who received ongoing supervision, conducted the interviews at the home visit. The interviews were recorded to later ensure inter-rater reliability coding by a coder blind to diagnostic status. Interrater reliability levels were excellent (90.2%, κ = .89 (*p* < .001)) as reported by Smith-Nielsen et al. [81].

To assess PD, we used the Standardized Assessment of Personality Abbreviated Scale (SAPAS) [82], a screening interview measuring disordered personality traits. The interview contains eight items that entail statements about the person concerning difficulties with social competencies, trust, emotion regulation, impulsivity, worrying and perfectionism. Based on the person’s response, each statement is coded as either 0 = “no” or 1 = “yes”. A score of 3 or more indicates probability of the presence of a PD, and “3” is thus the standardized cut-off of the SAPAS [82]. Studies have demonstrated a high correlation between the SAPAS and the Structural Clinical Interview for DSM-IV Axis II PDs [82,83]. A trained clinical psychologist administered the SAPAS at the home visit. A coder with no prior knowledge of the mother coded 20% of the recorded interviews. Inter-rater reliability was excellent (94.5%, κ = .88, *p* < .001).

The Hopkins Symptom Checklist (SCL) [84] was used to assess non-specific psychological distress. The SCL is a well-established questionnaire assessing psychological symptomatology across multiple domains and can be used to determine risk for one or more clinical disorders. We used the SCL with 63 items based on Olsen et al.’s [69] validation study in a Danish population sample of 1153 adults. The SCL-63 measures symptoms relating to psychiatric conditions on six subscales (anxiety, somatization, obsession–compulsion, interpersonal sensitivity, depression, and phobic anxiety). The composite Global Severity Index (GSI) measures current overall psychological distress, and Danish norms and gendered cut-off scores for the GSI-63 have been established in a population sample of 2040 adults [85], with a cut-off of 1.08 in GSI-63 raw score indicating clinical levels of mental distress in women. Cronbach’s alpha for the GSI-63 was .96.

### Statistical analyses

All analyses were conducted using IBM® SPSS® Statistics version 26.0. IBM® SPSS® AMOS® version 26.0 was used for factor analyses. To investigate the factor structure of the PRFQ, CFA was conducted in the PPD group (*n* = 237) and the no-PPD group (*n* = 186) separately using maximum likelihood estimation to examine the fit of the model in both groups. The fit indices used for the CFA were the χ^*2*^-test statistics (χ^2^/df), comparative fit indices (CFI), non-normed fit index (NNFI) and root mean square error of approximation (RMSEA). For a model to have an acceptable fit, χ^2^/df should be ≤ 3, CFI and NNFI should be > .90 and RMSEA should be < .08. With χ^2^/df values < 2 and RMSEA < .05, the model has an excellent fit [86,87].

Next, to investigate measurement invariance, multi-group CFA (MGCFA) with maximum likelihood estimation was conducted in the total sample of 423 mothers (PPD diagnosis, *n* = 237, no PPD diagnosis, *n* = 186) to test the equivalence of the factor structure of the PRFQ across mothers with and without diagnosed PPD. The fit indices used for overall model data fit in the MGCFA were the same as for the CFA. Following Putnick and Bornstein [32], we compared the unconstrained model (M0) to a model with constrained factor loadings to test for metric invariance (M1), and then compared the model with constrained factor loadings to a model with constrained factor loadings and intercepts (M2) to test for scalar invariance. As the χ^*2*^ value has been found to be sensitive to sample size [88], we used additional model fit indices to evaluate measurement invariance. Hence, we applied the following cut-off criteria as suggested by Chen [89] for samples with unequal group sizes: 1) ΔCFI ≤ –.005 and 2) ΔRMSEA ≥ .010 for loading invariance for both loading and intercept invariance, as well as overall model fit indications [90]. Finally, internal consistency was examined using Cronbach’s alpha.

Correlations between the PRFQsubscale scores and psychopathology measures were assessed using different coefficients: 1) Pearson’s product moment correlation coefficient for correlation between PRFQ scores, 2) Point-biseral correlation coefficient for correlation between PRFQ scores, PPD diagnosis (yes/no), SAPAS and SCL-63 GSI (above/below cut-off), and 3) phi correlation for correlation between PPD diagnosis (yes/no), SAPAS and SCL63-GSI (above/below cut-off).

To investigate the associations between PRF and PPD with or without comorbidity, multinomial logistic regression analyses were performed on a subsample of 344 mothers for whom both SAPAS and SCL-63 scores were available. In order to differentiate between mothers with and without diagnosed PPD and to also account for comorbidity with PPD, mothers were allocated to five groups based on clinical status: 1) subclinical mothers (no diagnosis of PPD, SAPAS and SCL-63 GSI below cut-off, *n* = 117), 2) mothers with only PPD (diagnosis of PPD, SAPAS below cut-off, SCL-63 GSI below cut-off, *n* = 38), 3) mothers with PPD and symptoms of PD (diagnosis of PPD combined with SAPAS above cut-off, *n* = 18), 4) mothers with PPD and clinical levels of psychological distress (diagnosis of PPD combined with SCL-63 GSI above cut-off, *n* = 79), and 5) mothers with PPD, symptoms of PD and clinical psychological distress (diagnosis of PPD combined with both SAPAS and SCL-63 GAI above cut-off, *n* = 92). As educational level is proposed to be associated with reflective thinking [91], we added maternal educational level as a covariate, which was grouped according to the International Standard Classification of Education by UNESCO (ISCED).

A post-hoc multinomial logistic regression was performed to investigate the relationship between PRF and symptoms of PD and clinical levels of psychological distress in mothers without the presence of PPD (*n* = 176). For this, mothers were classified by the following grouping variable: 1) both SAPAS and SCL-63 GSI below cut-off (*n* = 117), 2) SAPAS above cut-off, SCL-63 GSI below cut-off (*n* = 20), 3) SCL-63 GSI above cut-off, SAPAS below cut-off (*n* = 23), and 4) both SAPAS and SCL-63 GSI above cut-off (*n* = 16). Here, we also controlled for maternal educational level.

Prior to analysis, data were examined for outliers and normality. Based on *z*-scores [92], few outliers were identified on the PM scale (*n* = 3) and the IC scale (*n* = 5). Qualitative inspection of these cases indicated that the scores reflected valid responses on a Likert scale, and they were therefore not omitted from the analyses. As the PM and IC scales showed non-normal distribution, we ran correlations and the multinomial logistic regression with log-transformed PRFQ scales.

Following the proposed rules for evaluating adequate effect sizes, suggesting a ratio of 10 participants per variable item [88,93], the study sample size is sufficient for CFA and MGCFA. We conducted a post-hoc logistic regression power analysis based on the values in this sample to determine the minimum sample size if we wanted to replicate these findings in order to answer the main hypothesis, which was whether the PRFQ subscales could distinguish between PPD and no-PPD groups. For an odds ratio (OR) of 2.23, which was the lowest significant OR observed in the present study, a minimum sample size of 98 in total was needed to reach 80% power with a significance level of .05.

## Results

### Sample descriptives

Sample characteristics are presented in Table 1. As shown in Table 1, in general, this is considered a well-resourced sample. The majority of mothers were of Danish ethnicity (PPD group = 84.8%, no-PPD group = 84.9%), and were overall well educated, with an educational level equivalent to bachelor’s degree or higher (PPD group = 78.5%, no-PPD group = 75.3%). Concerning depression history, a greater percentage of mothers who currently fulfilled criteria for PPD had also experienced major depressive disorder previously, compared with mothers without current PPD (PPD group = 61.6%, no-PPD group = 45.2%). Most of the mothers had an infant between 0 and 5 months of age, with a mean age of 3.2 months (SD = 2.1) in the PPD group and 2.9 months (SD = 1.6) in the no-PPD group [88].

**Table 1:** Sample characteristics (n = 423).

Variable	PPD (*n* = 237, 56%)		No PPD(*n* = 186, 44%)	
Maternal age, mean (*SD*)	32.3	(4.6)	31.4	(4.6)
Range	22–47		22–44	
Missing, *n* (%)	1	(0.4)	0	
Infant age in months, mean (*SD*)	3.2	(2.1)	2.9	(1.6)
Range	1–11		1–9	
0–5 months, *n* (%)	207	(87.3)	170	(91.4)
6–11 months, *n* (%)	30	(12.7)	16	(8.6)
Infant gender, male, *n* (%)	124	(52.3)	101	(54.3)
Maternal ethnicity				
Danish, *n* (%)	201	(84.8)	158	(84.9)
Immigrant, *n* (%)	24	(10.1)	16	(8.6)
Descendant of immigrants, *n* (%)	2	(0.8)	5	(2.7)
Missing, *n* (%)	10	(4.2)	7	(3.8)
Relationship status, married/living with partner, *n* (%)	212	(89.4)	169	(90.8)
Not specified, *n* (%)	3	(1.3)	1	(0.5)
Missing, *n* (%)	10	(4.2)	7	(3.8)
Primiparous, yes (%)	154	(65)	137	(73.7)
Missing, *n* (%)	13	(5.5)	9	(4.8)
Maternal ISCED level of education				
Level 1–3 (lower secondary or less), *n* (%)	18	(7.6)	17	(9.1)
Level 4 & 5 (post-secondary, short-cycle tertiary), *n* (%)	24	(10.1)	20	(10.8)
Level 6 (bachelor or equivalent), *n* (%)	83	(35)	66	(35.5)
Level 7 & 8 (master + doctor or equivalent), *n* (%)	103	(43.5)	74	(39.8)
Missing, *n* (%)	9	(3.8)	9	(4.8)
Prior episodes of depression, *n* (%)	146	(61.6)	84	(45.2)
Missing, *n* (%)	4	(1.7)	1	(0.5)
SAPAS, mean (SD)	2.44	(1.43)	1.67	(1.21)
SAPAS above cut-off, *n* (%)	111	(46.8)	39	(21.0)
Missing, *n* (%)	4	(1.7)	3	(1.6)
SCL-63 GSI, mean (SD)	1.41	(0.54)	0.76	(0.4)
SCL-63 GSI above cut-off, *n* (%)	176	(74.3)	36	(19.4)
Missing, *n* (%)	0	0	3	(1.6)

*Notes*. PPD = Postpartum depression; ISCED = International Standard Classification of Education by UNESCO; SCL-63 GSI = Hopkins Symptom Checklist 63-items version General Severity Index [69]; SAPAS = Standardized Assessment of Personality, Abbreviated Scale [82].

### Confirmatory factor analysis

The initial model revealed a poor fit in both groups. In the PPD group (*n* = 237), item 10 did not load significantly on the PM scale. This was also the case for items 7 and 13 in the no-PPD group (*n* = 186). Based on these results, the three items with non-significant factor loadings were omitted, and a modified 15-item model was tested in both groups separately, referred to hereafter as the PRFQ-I, infant version. As shown in Table 2, the modified model had an excellent fit in the no-PPD group. After adding one error covariance between items 15 and 18 on the IC scale, the model also had an excellent fit in the PPD group. In the PPD group, PM correlated significantly with both CMS and IC (*r* = –.71, *p* < .001 and *r* = –.29, *p* = .014, respectively). Similarly, IC and CMS correlated significantly (*r* = .23, *p* = .006). There were no significant correlations between the PRFQ subscales in the no-PPD group. Factor loadings of the CFA are presented in Table 3. Mean PRFQ-I scores for the total sample of mothers (*n* = 423) were 2.03 (SD = 1.01) for the PM subscale, 3.71 (SD = 1.17) for the CMS subscale and 5.98 (SD = 0.81) for the IC subscale. Concerning scores specific to group status, mothers with no PPD ranged from 1 to 6.67 (*M =* 1.86, SD = 0.96) on the PM subscale, from 1 to 6.33 (*M* = 3.84, SD = 1.12) for the CMS subscale and from 1.67 to 7 (*M =* 5.99, SD = 0.78) for the IC subscale. For the PPD group, PM scores ranged from 1 to 5.33 (*M* = 2.16, SD = 1.03), CMS scores ranged from 1 to 6.5 (*M =* 3.62, SD = 1.19) and IC scores ranged from 1.83 to 7 (*M* = 5.97, SD = .83).

**Table 2:** Fit of the Confirmatory Factor Analysis (CFA) of the 15-item version of the Parental Reflective Functioning Questionnaire, Infant Version (PRFQ-I) with the total sample (n = 423), and measurement invariance (MGCFA) across groups (n = 237 mothers with PPD and n = 186 mothers without PPD).

	χ^*2*^	*df*	χ^*2*^*/df*	CFI	NNFI	RMSEA [90% CI]
CFA						
PPD	150.75**	74	1.753	.929	.913	.056 [.041, .071]
No PPD	122.08**	87	1.403	.935	.922	.047 [.025, .065]
MGCFA						
Unconstrained model (M0)	271.71**	172	1.580	.931	.918	.037 [.028, .045]
Constrained factor loadings (M1)	285.19**	184	1.550	.930	.922	.036 [.028, .044]
Difference M0 –M1	13.48	11	–	.001	–	.001
Constrained factor loadings and intercepts (M2)	314.69**	199	1.581	.920	.918	.037 [.029, .045]
Difference M1 –M2	29.5*	14	–	.010	–	-.001

*Note*. PPD = Postpartum depression; CFI = Comparative Fit Index; NNFI = Non Normed-Fit Index (also Tucker–Lewis Index, TLI); RMSEA = Root Mean Square Error of Approximation.

*p < .05

**p < .01.

**Table 3:** The 15-item infant version of the Parental Reflective Functioning Questionnaire (PRFQ-I) and standardized loadings from the confirmatory factor analysis, with mothers with PPD (left, n = 237), and mothers without PPD (right, n = 186).

Factor 1, “Certainty about Mental States”	Item 2	.84/.83
	Item 5	.72/.71
	Item 8	.67/.59
	Item 11 (R)	–.57/-.53
	Item 14	.43/.35
	Item 17	.79/.70
Factor 2, “Interest and Curiosity”	Item 3	.69/.78
	Item 6	.63/.58
	Item 9	.67/.54
	Item 12	.64/.61
	Item 15	.19/.40
	Item 18 (R)	–.43/–.26
Factor 3, “Prementalizing”	Item 1	.39/.35
	Item 4	.23/.19
	Item 16	.71/.98

*Note*. PPD = Postpartum depression. Reverse-scored items are denoted with (R).

### Multi-group CFA

Results of the MGCFA on the PRFQ-I are presented in Table 3. The MGCFA showed an excellent fit for the unconstrained model, indicating configural invariance, suggesting that the factor structure provided a good fit across the two groups. The CFI and RMSEA differences between models M0 and M1 were below the suggested thresholds, and the Δχ^*2*^ was insignificant (*p* = .335), indicating metric invariance, i.e. equivalence of factor loadings, indicating that each item contributes similarly to its latent construct across groups. When comparing the models M1 and M2 for scalar invariance testing, the ΔCFI and ΔRMSEA and overall model fit indices met the stated criteria; however, the Δχ^*2*^ was significant at the .05 level (*p* = .014). As the majority of the fit indices were within the specified limits, this suggested measurement invariance at the scalar level. At the scalar level, all item loadings were significant. In the PPD group, PM was negatively correlated with both CMS (*r* = –.70, *p* < .001) and IC (*r* = –.30, *p* = .009). IC and CMS were positively correlated (*r* = .23, *p* = .005). In the no-PPD group, only PM and CMS were significantly correlated (*r* = –.43, *p* < .001). Cronbach’s alpha was .40 for PM, .69 for IC and .83 for CMS in the PPD group. In the no-PPD group, alpha values were .46 for PM, .67 for IC and .78 for CMS.

### Associations between maternal PRF and symptoms of psychopathology

Table 4 presents the results from multinomial logistic regression analysis in which maternal PRFQ scores were entered as predictors of clinical group, with the subclinical group as the reference category. The regression model was statistically significant, χ^*2*^ (24) = 61.18, *p* < .001. Regarding the PM subscale of the PRFQ, an OR of 3.47 (95% CI [1.04; 11.61]) indicated a significant increase (*b* = 1.24, *p* = .044) in the odds of having PPD combined with symptoms of PD for each unit increase in PM. A significant increase in odds of having PPD combined with clinical levels of psychological distress (OR = 2.23, 95% CI [1.11; 4.49], *b* = 0.80, *p* = .025) for each unit increase in PM was also found. Concerning the group of mothers with the greatest number of indicators of psychopathology, i.e. PPD, symptoms of PD and clinical levels of psychological distress, an OR of 3.90 (95% CI [1.96; 7.78]) indicated a significant increase (*b* = 1.36, *p* < .001) in the odds of belonging to this particular group for each unit increase in PM. A significant relation was also found for CMS, with an OR of 0.356 (95% CI [0.14; 0.92]) being indicative of a decrease in the odds (*b* = –1.03, *p* = .033) of having PPD, symptoms of PD and clinical levels of distress for each unit increase in CMS. Correlation between PRFQ-I subscales and psychopathology measures are provided as supplementary material (S1 Table).

**Table 4:** Multinomial logistic regression analysis of PRFQ-I scores on the presence of psychopathology in mothers with PPD compared with mothers with no PPD (n = 344), grouped.

	*b*	Wald χ^*2*^	df	*p*	OR	95% CI	
						LL	UL
**PPD**							
PM	0.52	1.31	1	.253	1.68	0.69	4.11
CMS	–0.46	0.55	1	.459	0.63	0.19	2.13
IC	–1.60	1.94	1	.164	0.20	0.02	1.93
**PPD and PD symptoms**							
PM	1.24	4.06	1	.044	3.47	1.04	11.61
CMS	1.94	3.49	1	.062	6.99	0.91	53.74
IC	–1.36	0.63	1	.428	0.26	0.01	7.33
**PPD and clinical psychological distress**							
PM	0.80	5.04	1	.025	2.23	1.11	4.49
CMS	–0.51	1.08	1	.299	0.60	0.23	1.58
IC	0.52	0.24	1	.624	1.68	0.21	13.37
**PPD, PD symptoms and clinical psychological distress**							
PM	1.36	14.96	1	< .001	3.90	1.96	7.78
CMS	–1.03	4.55	1	.033	0.36	0.14	0.92
IC	1.83	2.55	1	.110	6.21	0.66	5.92

*Notes*. PRFQ-I = Parental Reflective Functioning Questionnaire, 15-item infant version; PM = Prementalizing; CMS = Certainty about mental states; IC = Interest and curiosity; PPD = Postpartum depression; PD = Personality disorder; OR = Odds ratio.

### Post-hoc analysis

To explore the relationship between PRF and symptoms of PD and clinical levels of psychological distress, both separately and combined, a post-hoc multinomial logistic regression analysis was conducted in which maternal PRFQ-I scores were entered as predictors of clinical group, with the subclinical group as the reference category. The regression model was statistically significant, χ^*2*^ (19) = 45.84, *p* < .001. Regarding the PM subscale of the PRFQ-I, an OR of 6.29 (95% CI [2.17; 18.20]) indicated a significant increase (*b* = 1.84, *p* = .001) in the odds of having clinical levels of psychological distress for each unit increase in PM. A significant decrease in the odds of having both symptoms of PD and clinical levels of psychological distress (OR = .07, 95% CI [0.01; 0.34], *b* = –2.74, *p* = .001) for each unit increase in CMS was also found. There was no significant relation between the IC subscale and any of the clinical groups. Results from the post-hoc analyses are provided as supplementary material (S2 Table).

## Discussion

The purpose of the present study was twofold. First, we investigated whether the proposed three-factor structure of the Danish PRFQ could be confirmed in a large sample of subclinical and clinical mothers of infants, and we investigated measurement invariance between mothers meeting diagnostic criteria for PPD and mothers not meeting diagnostic criteria for PPD. Second, we investigated the association between PRF and maternal psychopathology in mothers with diagnosed PPD, with and without clinical comorbid symptomatology, compared with mothers with subclinical symptoms of psychopathology.

We investigated these issues in a sample of mothers with infants aged 1–11 months. This was an urban sample, as the study was conducted as part of a collaborative research project with the public health visitors of the municipality of Copenhagen. The majority of mothers were well educated, of Danish ethnicity and currently in a relationship with a partner/father of the child. All mothers had been referred to the project based on screening for symptoms of PPD, and 237 mothers (56%) fulfilled diagnostic criteria for a diagnosis of major depression. In addition, in both the PPD and no-PPD groups of mothers, many were assessed for whether they met criteria for major depression prior to the present episode (PPD group = 61.6%, no-PPD group = 45.2%), indicating that for both the clinical and the subclinical group, depressive symptoms were a recurrent issue, which has also been demonstrated in previous studies [54,94]. Thus, despite being an otherwise low-risk sample, mothers in the study tended to have a range of clinical problems.

### Confirmatory factor analysis of the PRFQ and multi-group analysis of the PRFQ-I

Overall, the results confirmed a multidimensional, three-factor structure of the PRFQ in a modified model consisting of 15 items for mothers of infants, named the PRFQ-I for this study. Additionally, the invariance across mothers despite PPD diagnosis was confirmed at the scalar level. CFA of the original 18-item model revealed a poor model fit. By omitting items with insignificant factor loadings (items 7, 10 and 13 on the PM scale), the 15-item PRFQ-I was obtained. CFA of the PRFQ-I resulted in an excellent model fit in both the PPD and the no-PPD group. Group comparison with MGCFA resulted in configural, metric and scalar invariance, with an excellent fit at all three levels, indicating that mothers interpreted the items and latent constructs of the PRFQ-I in similar ways irrespective of diagnostic status [32]. In the constrained intercept model, the Δχ^*2*^ value was significant at the .05 level. However, chi-square value has been found to be sensitive to sample size, resulting in falsely rejecting adequate models when the sample size is large, and thus additional goodness-of-fit measures for invariance testing are suggested, such as ΔCFI and ΔRMSEA [88,89]. Nevertheless, as this is the first study on the PRFQ-I, future studies applying the PRFQ-I are recommended to examine it in terms of psychometric properties and measurement invariance.

We were able to confirm a three-factor structure of the PRFQ-I, which is in agreement with previous studies [23–25], although these studies made use of an 18-item version of the PRFQ. The discrepancy in models between the current study and previous findings might be due to considerable differences in samples, regarding both the clinical status of study participants and the age of their children. For instance, in our study, mean age child was 3.2 months in the PPD group and 2.9 months in the non-PPD group. In comparison, mean age child was 19 months in Luyten et al.’s [23] study and 6.72 years in the study by Pazzagli et al. [25], while in the study by Mousawi and Bahrami Ehsan [24] child age ranged from 1 to 5 years. As the capacity to mentalize is considered both relationship- and context-specific [1,95], it may be suggested that our version of the PRFQ better captures PRF specifically related to infancy, which might, however, alter during the course of parenthood.

It could be argued that the rather low internal consistency values for the prementalizing scale in mothers with and without diagnosed PPD reflects low reliability of this scale. However, as alpha values highly depend on the number of items in the assessed scale, values will increase as the number of items increases without correctly reflecting the reliability of the scale [96]. Furthermore, although general guidelines for alpha values exist, it has been suggested that when dealing with psychological constructs, lower alpha values can be expected, and even more so in early stages of research [97,98]. A scale with few items, in this case three items, should therefore not be rejected based on alpha values alone. Another point regarding the apparent low internal consistency of the PM subscale might concern the theoretical characteristics of the construct. Prementalizing is a complex dimension, with nuances in ways of expressing a prementalizing stance, which may vary across parents with impaired PRF. For instance, a parent could display a teleological mode of mentalizing, that is, an extreme exterior focus [10], which on the PRFQ and PRFQ-I is consistent with the statement “The only time I’m certain my child loves me is when s/he is smiling at me” (item 1). On the other hand, the statement “My child cries around strangers to embarrass me” (item 4) taps into a more pretend mode of mentalizing, where mental states are disentangled from reality. Finally, a complete repudiation or disavowal of reflective functioning is reflected in the statement “Often, my child’s behavior is too confusing to bother figuring out” (item 13) [10]. From this, is it possible for a parent to display one aspect of PM (e.g. teleological mode) but not express disavowal of mentalizing *per se*, and that the variety of these differentiated types of PM causes the low internal consistency of this particular subscale.

### Associations between maternal PRF and PPD

Findings on the association between PRF on the PRFQ-I and maternal psychopathology were mixed. No significant associations between impaired PRF and PPD were found; however, significant associations between PRF impairments and maternal psychopathology were found in mothers with both PPD and other comorbid symptoms of psychopathology, indicating that PRF capacities differed among mothers with respect to the severity of psychological difficulties.

Contrary to our expectations, a diagnosis of PPD was not associated with impaired PRF compared with mothers with no diagnosis of PPD. To our knowledge, only one other study has investigated PRF using the PRFQ in a sample of PPD-diagnosed mothers of young infants [47]. Unlike our findings, Krink et al. [47] reported that higher levels of depressive symptoms were associated with more prementalizing. However, that study differs from the current study in terms of sample: it included mothers with diagnosed mood disorder, and measured and reported current symptoms of depression by a self-report screening tool, the Beck Depression Inventory (BDI) [99]. Mothers with PPD are considered to be a quite heterogeneous group [100], and differences in time of onset, previous history of depressive episodes, symptoms and degree of severity may add to diversities in PPD profiles, implying variance regarding the relation between PRF and PPD [101]. It might be that severity of depression is more easily expressed with a symptom rating scale and that screening scores reflect a more prevailing and acute depressive state as opposed to diagnostic categories, thus having a more concurrent and critical impact on mentalizing abilities.

Another potential explanation for our findings could be related to sample characteristics. Some studies have found that the association between maternal PPD and adverse parenting practices is less prominent in samples with socio-economic advantages [102,103]. As the mothers in the current study are overall well resourced, other results might be found in higher-risk samples. In addition to this, all mothers in this study were referred to the study based on an initial positive screening for depression on the EPDS, and therefore we compared a clinical group with a subclinical group (as opposed to a non-clinical control group), and thus differences in PRF between mothers with and without PPD might not be as pronounced as those found in previous studies [47,48]. Similarly, some studies assessing psychosocial functioning in clinically depressed and subclinical individuals have reported few to no significant differences between groups [104,105].

### Associations between maternal PRF and PPD with comorbid symptoms of psychopathology

In accordance with our expectations, mothers with PPD combined with symptoms of PD or with clinical levels of non-specific psychological distress showed significantly more prementalizing towards their infant, compared with the subclinical group. The PM scale of the PRFQ-I represents both the pretend mode of mentalizing, in which the interpretation of internal states is separated from reality (e.g. “my child cries around strangers to embarrass me”), the teleological mode, in which interpretation is based solely on external behavior (e.g. “The only time I’m certain my child loves me is when he or she is smiling at me”) and repudiation of PRF (e.g. “often, my child’s behavior is too confusing to bother figuring out”). Concerning the group of mothers diagnosed with PPD and having both symptoms of PD and clinical levels of psychological distress, results showed that, compared with the subclinical group, these mothers had higher levels of prementalizing as well as lower levels of certainty about mental states, that is, they were more likely to doubt their ability to interpret their infant’s mental state and also to be less certain about what their child wants, thinks and feels. While interest and curiosity in the infant’s mental state is essential for the parent’s inclination to explore the subjective world of the infant, lowered certainty about mental states and distorted attributions limits the further mentalizing process, which potentially affects the parent’s sensitive responsiveness to the infant’s needs [13]. The result that PRF impairments are related to maternal PPD only in cases where comorbidity is present corroborates findings from a growing body of literature focusing on the broader aspects of maternal psychological difficulties in the postpartum period, as opposed to depression only, and its implications on the emerging mother–infant relationship [55,56,72,74].

The fact that neither of the clinical groups of mothers showed impairments in IC may be explained by the nature of the IC dimension in combination with mothers’ characteristics. Interest and curiosity in the child’s mental state has been proposed to be a core feature of PRF, as well as a more adaptive one [1,27,106]. It is therefore possible that in this well-resourced sample, mothers are indeed curious about and attentive to their child’s mental states, and that this fundamental aspect of PRF is less likely to be impaired than may be the case in more disadvantaged populations [18]. On the other hand, these results also indicate that, although mothers with PPD and comorbid psychological difficulties express interest and curiosity in their infant’s thoughts and emotions, they are less prone to take on a mentalizing stance when it comes to interpreting such mental states (i.e. prementalizing) and to be overly uncertain about the infant’s mental state and/or interpretations of the infant’s signals (i.e. certainty about mental states). These results highlight the need for practitioners to be attentive to mothers’ mentalizing skills at multiple levels, as there might be underlying impairments with PRF beyond expressed interest in the infant’s social world, such as problematic misinterpretation of the infant’s behavior or hypomentalizing, that is, excessive uncertainty about the infant’s mental states.

### Associations between maternal PRF, PD symptoms and psychological distress

Interestingly, exploratory post-hoc analysis showed that clinical levels of psychological distress were significantly related to PM. Hence, higher levels of prementalizing increased the probability of having clinical levels of non-specific psychological distress, regardless of depression status. These findings are similar to those of Luyten et al. [23], who also reported a correlation between maternal symptomatic distress and prementalizing. Our results indicating that general symptom severity, but not PPD, is associated with poorer PRF may be explained by the fact that the GSI cut-off reflects the demarcation between normal distress and psychiatric cases on a more dimensional global scale as opposed to a categorical diagnostic system such as the DSM-5. This is supported by Sandanger et al. [107], who compared a 25-item version of the SCL with a diagnostic interview (the Composite International Diagnostic Interview) [108] and found that the GSI of the SCL served as a better indicator of general caseness status, that it was more sensitive to different aspects of psychological suffering, and that clinical levels on the GSI of the SCL seemed to reflect a more urgent expression of distress.

Although no relation was found between symptoms of PD and PRF, post-hoc findings revealed that increased scores on the CMS subscale significantly decreased the odds of having both symptoms of PD and clinical levels of psychological distress. The non-significant association between PRF and symptoms of PD is contrary to previous findings [66,109,110]. From a mentalizing theory perspective, this finding might be explained by the dynamic nature of mentalizing skills [19]. While some evidence indicates that mentalizing entails trait-like features, it is nevertheless a capacity largely influenced by contextual circumstances, such as high arousal or stress [10]. Thus, the results indicate that PRF skills are primarily affected in overall well-functioning mothers with PD symptoms who are also experiencing elevated levels of psychological distress. Additionally, these mixed findings may also be due to both methodological issues and issues concerning differentiation in psychosocial functioning in mothers with symptoms of PD. Although it is highly correlated with diagnostic measures of PD [82,83], the SAPAS is not a clinical assessment but a screening tool, and the final score does not reflect a specific type of PD. It is likely that the mothers in this study present with symptoms of PDs across PD clusters, and that the types of PD vary regarding their severity and pervasiveness, and thus differ in terms of the level of mentalizing impairments and the domains of mentalizing that are affected [111,112].

### Implications

The findings from the present study indicate that when examining PPD as a risk factor, for example, for PRF, as we do in this study, it is important to look beyond the symptoms of PPD and to focus on other risk factors associated with PPD and potential stressors following childbirth, such as general symptom severity and symptoms of PD. By confirming a three-factor structure of the PRFQ-I and demonstrating measurement invariance, the findings add to current research on the PRFQ as a valid and feasible self-report measure of PRF. Our results are also in line with the growing body of literature on PRF as a multidimensional construct, with the different modes being related to variability in maternal psychological functioning. Overall, as our results suggest that PRF impairments are more pronounced with co-occurring psychological problems, awareness of other symptomatology, such as general psychological distress and symptoms of PD, might provide further insight into which mothers may be at risk for having impairments in their parental reflective capacities, and which may not. In addition to this, using a multidimensional approach when assessing PRF can help to tailor the focus of treatment to fit the needs of individual mothers based on their strengths and difficulties regarding specific aspects of mentalizing [23]. The PRFQ-I has the potential to be implemented in both research and early maternal mental health care practices in Denmark, as a preliminary assessment and screening tool for PRF in mothers, and to be added to the methods used to identify mothers with postpartum psychological difficulties. Finally, although this study examines PRF in association with psychopathology only, the results tap into the notion of PRF as a complex and dynamic capacity at interplay with variability in context [113]. This further puts emphasis on the value of applying a multidimensional approach to the assessment of PRF, and on how the dimensions interact differently with important factors, such as the influence of partners [114,115] and the social environment [116], as well as the parent’s experiences with their own caregiver(s) [15,27,117].

### Limitations and future directions

All participating mothers were referred for the project based on scoring above the cut-off in a screening with the EPDS, and therefore being considered to be at-risk. However, the sample was an overall well-resourced urban sample, which limits generalizability of our results to higher-risk populations. The well-resourced nature of the sample was partially due to the overall project inclusion criteria, which excluded mothers with higher-risk characteristics such as substance abuse and severe mental disorder. Further, mothers were quite homogenous in terms of demographic features such as educational level and relationship status. In addition, although we divided mothers into a PPD and a no-PPD group based on diagnostic assessment, the no-PPD group in this study cannot be considered a healthy control group, but rather a subclinical group. Further studies with more disadvantaged samples and/or using non-clinical controls as the comparison group are therefore needed. Another limitation is that the actual conducted analyses were multinomial logistic regressions, which need more power due to their categorical nature, and some of the subgroups were relatively small, which may have impacted the power to detect significant results in distinguishing these subgroups.

This study addressed the relationship between PRF and different aspects of symptoms of maternal psychopathology; however, being a cross-sectional study, the results do not address the causal relationship between PRF and maternal psychopathology. Thus, future studies using longitudinal study designs are needed. Considering that PRF is proposed to be influenced by contextual factors [95], it would also be highly relevant to further investigate PRF as a dynamic function that evolves with the developing child and the parent–infant relationship. This calls for future studies that apply the PRFQ to examine the factor structure at different time points in parenthood in order to make valid assumptions about the role of PRF in parenting, track the potential development of PRF, and compare groups in a valid way [32].

As a self-report measure, the PRFQ may be influenced by a number of biases, such as social desirability or psychological defenses that potentially distort mothers’ self-evaluation of their mentalizing capacities and their authentic response [118]. The gold-standard assessment of PRF skills is thought to be expert ratings based on the Reflective Functioning Scale (RFS) for attachment interviews [19]. Although such interviews might also evoke defenses and social desirability biases, the scoring system has been developed to circumvent such responses and thus to overcome their influences on PRF rating. These biases might impact results of the factor analysis and thus the obtained factor structure and internal consistency values, as well as findings on the association between PRF and maternal psychopathology. In addition, while providing evidence for the sensitivity of the PRFQ-I to specific psychopathology profiles in mothers, this study does not include any assessment of the validity or reliability of the PRFQ-I with other measures of reflective capacities. However, a recent study has provided encouraging results on the convergent validity between the PRFQ and PRF assessed with the RFS applied to the Parent Development Interview, although this was conducted in a low-risk, non-clinical sample [119].

Furthermore, initial data screening showed that the PM and IC subscales were non-normally distributed, indicating that there was little variance in mothers’ responses on these two scales. Further research might benefit from measuring PRF on the PRFQ in a non-linear manner, for example, by classifying parental responses into groups based on their PRFQ scores (low, medium, high), which may provide different results. Another issue with linearity of scores concerns the possibility of extreme scores on the CMS and IC subscales reflecting impaired PRF, i.e. hypermentalizing. Anis et al. [119] recoded the PRFQ CMS and IC subscales’ scores to account for high scores being potentially indicative of PRF deficits. By this approach, new scores were created based on deviations from the sample mean IC and CMS scores, and results supported the notion that, concerning the IC and CMS subscales, average scores of PRF may indicate better PRF. Although these results offer potentially valuable new ways to overcome issues with identifying impaired PRF on the CMS and IC subscales in future research, this approach could not be used in the present study, with reference to the aforementioned limitation of the scope of this study, i.e. the lack of assessment of the reliability and validity of the PRFQ with another measure of PRF. As optimal mid-range scores of the PRFQ subscales have been proposed to vary with sample characteristics, such as child age [23], validating the PRFQ-I against another measure of PRF would therefore be an important first step in determining whether the sample mean IC and CMS scores can indeed be considered indicative of the most optimal PRF.

## Conclusion

This study provides new evidence in relation to the multidimensional assessment of maternal self-reported reflective skills as measured by a modified infant version of the PRFQ, the PRFQ-I, as well as further steps towards a greater understanding of the differences in symptomatology associated with impaired PRF in mothers. Our findings indicate that maternal PPD *per se* might not be related to lowered PRF, and thus might not pose an unequivocal risk for the parent–infant relationship, but that impaired PRF is more likely to occur in mothers with a combination of symptoms of psychopathology, such as postpartum depression, symptoms of PD and overall psychological distress. However, additional research using the PRFQ-I in more diverse samples is needed to determine its general applicability and validity, and to further investigate the association between maternal psychopathology and PRF.

## Supporting information

S1 Table Correlations for study variables. (DOCX)

S2 Table Post-hoc multinomial logistic regression analysis of PRFQ-I scores on the presence of symptoms of personality disorder and clinical levels of psychological distress (n = 176), grouped. (DOCX)
