# Supplementary material for: Factor structure of the parental reflective functioning questionnaire and association with maternal postpartum depression and comorbid symptoms of psychopathology
Source: PLoS One. 2021 Aug 2;16(8):e0254792. doi: 10.1371/journal.pone.0254792 (PMC8328297; doi:10.1371/journal.pone.0254792)
Supplement: S1 Table — (DOCX) [file pone.0254792.s001.docx]

| **S1 Table.**  *Correlations for study variables* | | | | | |
| --- | --- | --- | --- | --- | --- |
| Variable | 1 | 2 | 3 | 4 | 5 |
| 1. PM | - |  |  |  |  |
| 2. CMS | -.38**^a^ | - |  |  |  |
| 3. IC | -.17**^a^ | .09^a^ | - |  |  |
| 4. PPD | .16**^b^ | -.11^b^ | -.01^b^ | - |  |
| 5. SAPAS | .13**^b^ | -.09^b^ | .03^b^ | .27**^c^ |  |
| 6. SCL63-GSI | .23**^b^ | -.20**^b^ | .06^b^ | .52**^c^ | .32**^c^ |
| *Note.* PM = Prementalizing; CMS = Certainty about mental states; IC = Interest and curiosity; PPD = postpartum depression; SAPAS = Standardized Assessment of Personality, Abbreviated Scale; SCL63-GSI = Hopkins Symptom Checklist 63-items version General Severity Index.  *p < .05, **p < .01  ^a^Pearson’s product moment correlation coefficient  ^b^Point-biseral correlation  ^c^Phi correlation coefficient | | | | | |
