# Supplementary material for: Factor structure of the parental reflective functioning questionnaire and association with maternal postpartum depression and comorbid symptoms of psychopathology
Source: PLoS One. 2021 Aug 2;16(8):e0254792. doi: 10.1371/journal.pone.0254792 (PMC8328297; doi:10.1371/journal.pone.0254792)
Supplement: S2 Table — (DOCX) [file pone.0254792.s002.docx]

| **S2 Table.**  *Post-hoc multinomial logistic regression analysis of PRFQ-I scores on the presence of symptoms of personality disorder and clinical levels of psychological distress (n = 176), grouped* | | | | | | | |
| --- | --- | --- | --- | --- | --- | --- | --- |
|  | *b* | Wald χ*^2^* | df | *p* | OR | 95% CI | |
|  |  |  |  |  |  | LL | UL |
| **PD** |  |  |  |  |  |  |  |
| PM | 0.46 | 0.59 | 1 | .441 | 1.59 | 0.49 | 5.12 |
| CMS | -0.45 | 0.91 | 1 | .620 | 0.64 | 0.11 | 3.76 |
| IC | -0.85 | 0.42 | 1 | .518 | 0.43 | 0.03 | 5.68 |
| **Clinical psychological distress** |  |  |  |  |  |  |  |
| PM | 1.84 | 11.49 | 1 | .001 | 6.29 | 2.17 | 18.2 |
| CMS | -0.77 | 1.0 | 1 | .318 | 0.46 | 0.10 | 2.09 |
| IC | 1.03 | .33 | 1 | .563 | 2.81 | 0.09 | 93.17 |
| **PD & clinical psychological distress** |  |  |  |  |  |  |  |
| PM | 0.52 | 0.62 | 1 | .430 | 1.68 | 0.46 | 6.09 |
| CMS | -2.74 | 10.52 | 1 | .001 | .07 | 0.01 | 0.34 |
| IC | 2.91 | 1.11 | 1 | .293 | 18.27 | 0.08 | 4105.15 |
| *Notes.* PRFQ-I = Parental Reflective Functioning Questionnaire, 15-item infant version; PM = Prementalizing; CMS = Certainty about mental states; IC = Interest and curiosity; PD = Personality disorder; OR = Odds ratio*.* | | | | | | | |
